# Quantitative Assessment of Blood Pressure Measurement Accuracy and Variability from Visual Auscultation Method by Observers without Receiving Medical Training

**DOI:** 10.1155/2017/3537079

**Published:** 2017-12-20

**Authors:** Wenai Chen, Fei Chen, Yong Feng, Aiqing Chen, Dingchang Zheng

**Affiliations:** ^1^Department of Cardiology, Second Affiliated Hospital, School of Medicine, Zhejiang University, Hangzhou, China; ^2^Department of Electrical and Electronic Engineering, Southern University of Science and Technology, Shenzhen, China; ^3^Department of Medical Science and Public Health, Faculty of Medical Science, Anglia Ruskin University, Chelmsford, UK

## Abstract

This study aimed to quantify blood pressure (BP) measurement accuracy and variability with different techniques. Thirty video clips of BP recordings from the BHS training database were converted to Korotkoff sound waveforms. Ten observers without receiving medical training were asked to determine BPs using (a) traditional manual auscultatory method and (b) visual auscultation method by visualizing the Korotkoff sound waveform, which was repeated three times on different days. The measurement error was calculated against the reference answers, and the measurement variability was calculated from the SD of the three repeats. Statistical analysis showed that, in comparison with the auscultatory method, visual method significantly reduced overall variability from 2.2 to 1.1 mmHg for SBP and from 1.9 to 0.9 mmHg for DBP (both *p* < 0.001). It also showed that BP measurement errors were significant for both techniques (all *p* < 0.01, except DBP from the traditional method). Although significant, the overall mean errors were small (−1.5 and −1.2 mmHg for SBP and −0.7 and 2.6 mmHg for DBP, resp., from the traditional auscultatory and visual auscultation methods). In conclusion, the visual auscultation method had the ability to achieve an acceptable degree of BP measurement accuracy, with smaller variability in comparison with the traditional auscultatory method.

## 1. Introduction

Hypertension is the third leading cause of death worldwide [1]. Cardiovascular disease causes 17 million deaths per annum globally with complications arising from hypertension accounting for 9.4 million [2]. Blood pressure (BP) measurement is the first important step in correctly diagnosing hypertension and is one of the most common clinical skills that every medical professional needs to master [3].

There are currently two main noninvasive ways of measuring BPs: manual auscultatory technique and automatic oscillometric technique [4, 5]. Manual auscultatory BP measurement has been in use for over 100 years and has changed little over this time, which has been regarded as the most accurate and the gold standard noninvasive clinical BP measurement technique. It is also used as a reference technique for evaluating automated BP devices [6]. Manual auscultatory technique contains three main elements: a cuff, pressure display, and stethoscope [7–9]. The cuff encircles the upper arm to occlude the brachial artery and is deflated with a control, allowing the blood to flow again as the pressure is released. The pressure display has traditionally been mercury, but there are worldwide moves to ban mercury on environmental grounds, and the mechanical aneroid display is an alternative. The stethoscope is used to listen to the appearance and disappearance of Korotkoff sounds. Regarding the measurement principle of the manual auscultatory technique, systolic blood pressure (SBP) is defined when the Korotkoff sound appears for the first time during cuff pressure deflation, and diastolic blood pressure (DBP) is noted when the Korotkoff sound disappears.

The manual auscultatory BP measurement technique requires medical training and experience [10, 11]. Users often find the identification of systole and diastole by a stethoscope difficult. The measurement accuracy relies heavily on the skills of using a stethoscope to identify sounds associated with SBP and DBP, which could be affected by subjective factors, including the observers' auditory acuity and reaction speed. Therefore, manual auscultatory BP measurements are mainly performed by trained physicians and other health professionals.

However, the presence of a medical professional can cause artificially high BP [12, 13]. Measuring BPs at a nonclinical setting or in the home monitoring setting has therefore been recommended by some national hypertension societies for mitigating white coat effect [14, 15]. Automated devices are often used for self-measurement of BP because they are easy to operate. The majority of current automated BP devices are based on the oscillometric technique, where a pressurized cuff, as in the auscultatory technique, is wrapped around the upper arm, and pulses known as oscillometric pulses that are induced in the cuff are captured and mathematically modelled to determine SBP and DBP [16–18]. Unfortunately, automated oscillometric technique only estimates and does not truly measure BPs since these devices are typically calibrated against the average values from a group of subjects using characteristics ratios, not for individual subject, resulting in that their accuracy is not adequate for many clinical diagnostic decisions [19]. The current international standards show that automated BP devices can have an inaccuracy of +/−16 mmHg (95% confidence interval) in comparison with manual auscultatory measurement [6].

From the measurement principle point of view, the traditional manual auscultatory method using stethoscope can measure actual BPs for individual subject. Instead of listening for the Korotkoff sounds, visualizing the digitally recorded Korotkoff sound waveform during cuff deflation for BP determination provides an alternative to the traditional manual method [20, 21]. However, the BP measurement accuracy and variability from visualizing Korotkoff sound waveform have not fully quantified. This study aimed to provide scientific evidence on these data, with a focus on the observers without receiving medical training, allowing the competence of performing visual auscultation BP measurement by the general public at home to be assessed.

## 2. Methods

### 2.1. Database of Korotkoff Recordings

The online educational BP measurement training database from the British Hypertension Society (BHS, http://bhsoc.org/resources/bhs-dvd/) was used in this study [22]. All the BP measurements in the database strictly followed the manual auscultatory BP measurement guidelines with the subjects at the sitting position and their arms supported at the level of the heart [7]. The database is commonly used for mastering manual auscultatory BP measurement skills and assessing measurement competence. It includes 32 video clips of Korotkoff sound recordings, each of which shows a mercury column whilst a manual auscultatory BP measurement is being taken. Two of them are duplicated (T6 repeats T1, and T31 repeats T27). A total of 30 recordings were therefore used in this study, which cover a wide range of clinical situations, including recordings from health subjects, patients with different kinds of arrhythmia, and conditions that we frequently meet in our daily clinical work. The manual auscultatory BP reference answers are provided for each recording by the BHS, which were obtained by 24 experienced experts (see http://bhsoc.org/files/3913/4400/5764/Tutorial_answers_Erratum_Sept_09.pdf). Using the database of Korotkoff recordings with reference answers allowed the BP determinations from different techniques and observers to be compared.

### 2.2. Blood Pressure Measurement Observers

Ten BP measurement observers without any professional medical background were invited to determine BPs from the training database in a quiet assessment room. The observers without receiving medical training were specifically chosen as the secondary aim of the study was to assess the competence of the general public to perform auscultatory BP measurement with some simple instructions. The observers had normal hearing ability and had no hearing loss problems. This study has been reviewed and approved by the Human Research Ethics Committee of Southern University of Science and Technology. The investigation conformed with the Declaration of Helsinki, and all observers gave their written informed consent to participate in the study.

### 2.3. Blood Pressure Measurement Determinations

**Figure 1:**
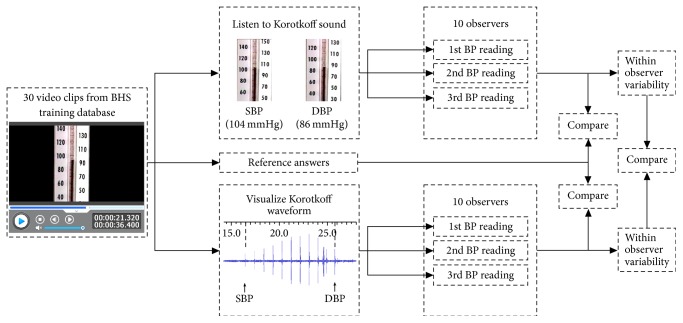
Blood pressure determination procedure by 10 observers using two measurement methods: traditional manual auscultatory method of listening for Korotkoff sounds and visual auscultation method by visualizing the Korotkoff sound waveform. The data analysis procedure is also given.

Figure 1 shows the experiment design and procedure. Each observer was asked to determine SBP and DBP using two measurement techniques: (a) traditional manual auscultatory method of listening for Korotkoff sounds and (b) visual auscultation method by visualizing the Korotkoff sound waveform. They are referred to below simply as the “traditional manual method” and “visual auscultation method,” respectively. Each observer was blinded to the reference answers. The observers were simply instructed to determine SBP and DBP by listening for or visualizing the appearance and disappearance of Korotkoff sounds, respectively. Before the formal BP determination, they were given the opportunity with some trials to be familiar with the procedure.

For the traditional manual method, the observers were asked to watch the mercury column from each of the 30 video clips, listen to the Korotkoff sounds during cuff pressure deflation, and make a note of the SBP and DBP they would determine. All the Korotkoff sounds from the 30 video clips were randomly played to each observer using Windows Media Player from the Microsoft Windows 8 (2013 Microsoft Corporation) and via an earphone (Lenovo in-ear headset P165). The same computer and earphone were used throughout the study. The computer volume was preadjusted and fixed to each observer. To mimic the BP measurement in clinical practice, each video clip was only allowed to replay once to each observer during the experiment. This same experiment procedure was repeated three times on different days with different randomised orders.

**Figure 2:**
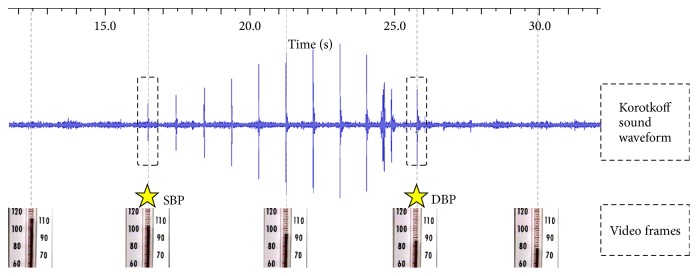
Illustration of converted Korotkoff sound waveform with some examples of video frames, and demonstration of SBP and DBP determination by visualizing the appearance and disappearance of Korotkoff sounds, respectively.

To implement the visual auscultation method, the audio signals from the 30 video clips were converted and digitalized to Korotkoff sound waveforms using software developed on MATLAB R 2012b (Mathworks, USA). Specially, the video.MultimediaFileReader object in MATLAB Image Processing and Video Toolbox was employed to extract the audio signals from each of the video clips. The sampling rate of the extracted audio signal was the same as that of the original video clip. A series of corresponding mercury column frames with a frame rate of 25 FPS were also produced from the beginning to the end of the video. As shown in Figure 2, the 10 observers were asked to mark the two timing points on the converted waveforms by visualizing the appearance and disappearance of Korotkoff sounds, from which the SBP and DBP were determined, respectively, from the two corresponding video frames with mercury column. Again, all the 30 Korotkoff sound waveforms were randomly presented to each observer, and three repeated readings were performed on different days by each observer.

### 2.4. Data and Statistical Analysis

As shown in Figure 1, there were 1800 BP values obtained in total, separately for SBP and DBP (from 30 recordings, 10 observers, 2 measurement techniques, and 3 repeated determinations). The overall mean and standard deviation (SD) of the measurement errors (difference between BPs determined by each observer and reference BPs) were calculated for all the Korotkoff recordings, separately for the two measurement techniques and for each observer. The histogram of BP measurement error was plotted, separately for the two measurement techniques.

Next, within-subject BP measurement variability was calculated from the SD of the three repeated measurements, respectively, for the BPs measured from the two techniques. A paired *t*-test was then performed to compare the measurement variability parameters between the two techniques. A value of *p* < 0.05 was considered a statistically significant difference.

Analysis of variance (ANOVA) was performed using the SPSS Statistics 19.0 software package (SPSS Inc., USA) to investigate the measurement repeatability of the three repeated BP determinations and the within-observer measurement variability between the two techniques. The intraclass correlation coefficient (ICC) was also obtained to study the between-observer effect. The statistical significance of measurement error in comparison with the reference answers was also determined, separately for the two measurement techniques, and for each observer.

## 3. Results

### 3.1. Measurement Repeatability and Within-Observer Variability

**Figure 3:**
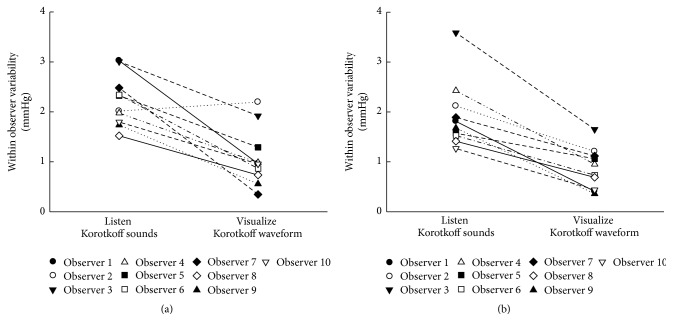
Within-observer measurement variabilities (SD of three repeated measurements on each Korotkoff recording) of SBP (a) and DBP (b) from the two measurement techniques: traditional manual auscultatory method of listening for Korotkoff sounds and visual auscultation method by visualizing the Korotkoff sound waveform. The average measurement variability across the 30 Korotkoff recordings is plotted individually for each observer.

Statistical analysis showed that there was no significant BP difference (for both SBP and DBP) between the three repeated determinations (*p* = 0.06 and 0.3 for SBP, and *p* = 0.42 and 0.20 for DBP, resp., for the traditional manual method and the visual auscultation method). Specifically, Figure 3 shows the comparison of within-observer measurement variability (SD of repeats in mmHg) for SBP and DBP between the two measurement techniques. It is shown that the visual auscultation method significantly reduced SD of repeats (*p* < 0.001 for both SBP and DBP), with an overall decrease across the 10 observers from 2.2 to 1.1 mmHg for SBP and from 1.9 to 0.9 mmHg for DBP. The decrease of within-observer variability was observed from each individual observer, except one SBP measurement from one observer.

### 3.2. Measurement Errors from the Two Measurement Techniques

**Figure 4:**
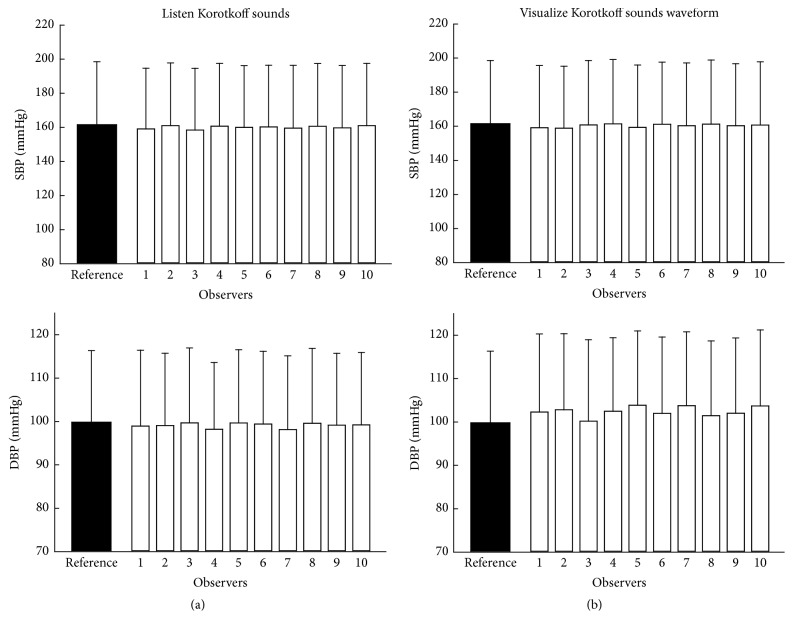
Mean and SD of SBP and DBP measured by each individual observer using the two measurement methods: traditional manual method (a) and visual auscultation method (b). The reference answers of SBP and DBP provided by the British Hypertension Society are also given.

**Figure 5:**
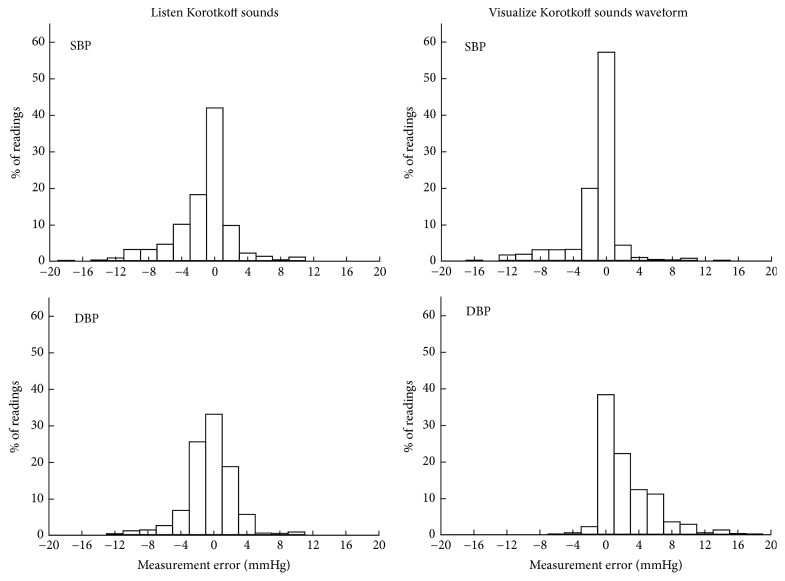
Histograms of measurement errors of SBP and DBP from each individual observer using the two measurement methods: traditional manual method and visual auscultation method. There are a total of 900 comparisons for each measurement method (from 30 Korotkoff recordings, 10 observers, and 3 repeated determinations).

Figure 4 shows the measured SBP and DBP by each individual observer using the traditional manual and the visual auscultation methods.  Figure 5 shows the histograms of SBP and DBP measurement errors, separately for the two measurement techniques. It is shown that 83% of SBP measurements and 90% of DBP measurements achieved a measurement error of less than or equal to 4 mmHg from the traditional manual method. Their corresponding values were 86% and 76% from the visual auscultation method, confirming that the visual auscultation method had the ability to achieve an acceptable degree of BP measurement accuracy.

**Figure 6:**
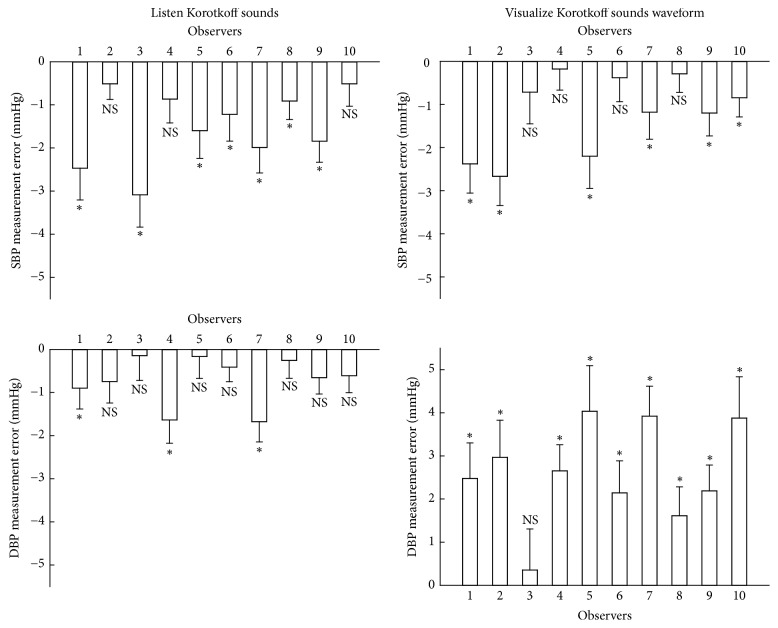
Mean and SEM of BP measurement errors of SBP and DBP from each individual observer using the two measurement methods: traditional manual method and visual auscultation method. Asterisk indicates that there is a significant difference between the measured BP values in this study and those reference answers provided by the BHS training database. “NS” indicates that there is no significant difference.

For both measurement techniques, statistical analysis from ANOVA and ICC showed that there was significant difference between observers in measured SBP and DBP (all *p* < 0.01).  Figure 6 shows the measurement error for each individual observer in detail, separately for the two measurement techniques. Overall, the best performance was observed from DBP measurement using the traditional manual method, where 7 out of 10 observers achieved nonsignificant measurement error in comparison with the reference answers. Statistical analysis also showed that the average DBP across the 10 observers measured from the traditional manual method was statistically comparable with the reference answers (*p* = 0.06), but there was significant difference in SBP for both measurement techniques and in DBP from the visual auscultation method (all *p* < 0.01).

Although significant, the overall means of SBP measurement error were small, which were −1.5 mmHg (between −0.5 and −3.1 mmHg across all the observers) and −1.2 mmHg (between −0.2 and −2.7 mmHg across all the observers), respectively, for the traditional manual auscultatory and visual auscultation methods. Their corresponding values for DBP measurement errors were −0.7 mmHg (between −0.1 and −1.7 mmHg across all the observers) and 2.6 mmHg (between 0.4 and 4.0 mmHg across all the observers), respectively, for the traditional manual auscultatory and visual auscultation methods.

## 4. Discussion and Conclusion

This study has quantified BP measurement errors from the traditional manual auscultatory method of listening for Korotkoff sounds and from the visual auscultation method by visualizing the Korotkoff sound waveform by observers without receiving professional medical training. It has been widely accepted that there are uncertainties for SBP and DBP determinations using manual auscultatory method, and there are differences in BP measurements between operators [23, 24]. Although there were significant systematic measurement errors of no more than 2 mmHg for both SBP and DBP, since all the measurements were performed by observers without receiving professional training who was only provided with very simple instructions on BP determination, the outcome of measurement accuracy should be considered to be satisfactory. With some added criteria on the BP determination rules and more specific instructions, increased measurement accuracy could be expected. Furthermore, each individual observer in this study achieved the required BP measurement accuracy according to the current international standards for BP device validation with the mean difference of no more than 5 mmHg and SD of difference no more than 8 mmHg in comparison with the manual auscultatory measurement by trained clinical observers, indicating that the general public has the potential ability and competence to perform clinical BP measurements using both methods with reasonable accuracy.

**Figure 7:**
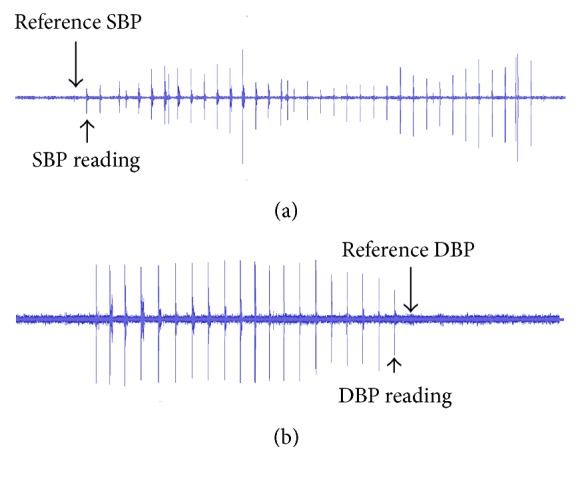
Waveform examples of Korotkoff sounds to illustrate the uncertainties of determining BPs from visual auscultation method, leading to BP measurement errors. The SBP and DBP were determined from one beat below and one beat above the actual audible Korotkoff sounds at systole and diastole, respectively.

The measurement errors are partially caused by the uncertainties of BP determination from the unclear Korotkoff sounds heard or from the unclear sound waveform visualized by the observer at systole and diastole [25].  Figure 7 illustrates the waveform examples for these uncertainties of BP determination from visual auscultation method. It can be seen that the actual audible Korotkoff sounds (from the reference BP readings) had very low amplitude, and the sound characteristic at systole and diastole may not be easily differentiated when compared with the non-Korotkoff sound beats. In future studies, with the application of advanced signal processing techniques, the originally recorded Korotkoff sounds could be converted into other visual representations, such as the spectrogram with the spectrum of frequencies of sound as they vary with time, which would help the actual Korotkoff sounds at systole and diastole to be more recognizable to the observers. In addition, with the combination of visual presentation of the sounds and the simultaneous replay of the audio sounds, better BP measurement accuracy could be expected.

More importantly, this study has shown that, with the visual auscultation method, within-observer measurement variability has been reduced significantly from 2.2 to 1.1 mmHg for SBP and from 1.9 to 0.9 mmHg for DBP in comparison with the manual auscultatory method of listing for the Korotkoff sounds. In particular, for these good quality Korotkoff sound waveforms, the same BP determination was always made from over 60% recordings with no measurement variability between repeats, indicating that the visual auscultation technique has very good measurement reliability. Furthermore, the visual auscultation method has effectively avoided some potential effects of subjective factors, such as reaction time, on BP determination, leading to smaller measurement variability.

In conclusion, the study has demonstrated that BP measurement using visual auscultation method could achieve an acceptable degree of accuracy by observers without receiving clinical training and achieve better measurement variability in comparison with traditional manual auscultatory method, providing scientific evidence that visual auscultation method could be implemented as a technique as self-measurement of BPs at nonclinical setting.
